# Evaluation of the Antimicrobial Efficacy of Ethanolic and Aqueous Extracts of Licorice (Glycyrrhiza glabra) Against a Periodontal Pathogen: An In Vitro Study

**DOI:** 10.7759/cureus.73789

**Published:** 2024-11-15

**Authors:** Hariharan S, Gayathri K, Ravishankar PL, Prem Blaisie Rajula, Sunanda Rao, Murali Venkata Rama Mohan Kodali, Kalaivani V, Saravanan A.V., Viola Esther, Supriti Brahma

**Affiliations:** 1 Periodontology, SRM Kattankulathur Dental College and Hospital, SRM Institute of Science and Technology, Chengalpattu, IND; 2 Oral and Maxillofacial Surgery, King Faisal University, Al Ahsa, SAU; 3 Periodontology, SRM Kattankulathur Dental College and Hospital, SRM Institute of science and technology, Chengalpattu, IND

**Keywords:** chlorhexidine, chronic periodontitis, extract, licorice, periodontal pathogen

## Abstract

Background: Periodontal disease is a major contributing factor to tooth loss and can significantly impact overall health. Traditional periodontal therapy typically involves mechanical debridement to remove plaque and calculus, along with the use of antimicrobial agents to reduce bacterial load. Licorice (*Glycyrrhiza glabra*) has a long history of use in traditional medicine for treating various ailments since it contains glycyrrhizin, glabridin, liquiritin, and flavonoids, which possess anti-inflammatory, antioxidant, and antimicrobial activities. Hence, licorice can be considered a viable alternative for managing periodontal inflammation.

Aim: The objective of this study is to conduct a comparative evaluation of the antimicrobial efficacy of ethanolic and aqueous extracts of licorice (*Glycyrrhiza glabra*) against a key periodontal pathogen, *Prevotella intermedia (P. intermedia)*

Methodology: In this study, aqueous and ethanolic preparation of licorice was done, and the antimicrobial effect of these extracts against *P. intermedia*was tested by evaluating the minimum inhibitory concentration (MIC). The gold standard chemical plaque control agent chlorhexidine was used as a control.

Result: Results of the present study showed that licorice extract has antimicrobial activity against *P. intermedia. *The MIC of aqueous and ethanolic licorice root extracts against *P. intermedia *was established at 0.8% and 0.1%, respectively. When the aqueous and ethanolic extracts were compared, the ethanolic extract of licorice showed significantly more antimicrobial properties than the aqueous extract.

Conclusion: Ethanolic extract of licorice possesses highly significant antibacterial activity against *P. intermedia*; hence, it can be used as an alternative to chlorhexidine mouthwash.

## Introduction

Periodontal disease is a major contributing factor to tooth loss and can significantly impact overall health, as it is correlated to systemic conditions, most commonly diabetes and cardiovascular and respiratory infections [1]. Periodontal disease is primarily characterized by chronic plaque accumulation in susceptible hosts. The primary etiological agents of periodontal diseases are pathogenic bacteria, notably *Porphyromonas gingivalis *(*P. gingivalis*), *Aggregatibacter actinomycetemcomitans *(*A. actinomycetemcomitans*), *Tannerella forsythia* (*T. forsythia*), *Treponema denticola *(*T. denticola*), and orange complex bacterial species like *Prevotella intermedia* (*P. intermedia*) and *Fusobacterium nucleatum *(*F. nucleatum*). These microorganisms thrive in subgingival biofilm and elicit an inflammatory response that leads to the destruction of periodontal tissues [2]. Further, the presence of *P. intermedi*a has been positively correlated with clinical attachment loss, bleeding on probing, and periodontal inflammation [3]. *Prevotella intermedia* is a Gram-negative, anaerobic, spore-forming, and non-motile bacteria. They form a fraction of the oral flora, colonizing the oral cavity, and turn into pathogenic species with the increase in their number. These microorganisms were found to be getting higher in subgingival plaque in areas with periodontal disease associated with hormonal changes [4]. Invasion of periodontal tissues by these bacteria leads to an increase in levels of matrix metalloproteinases (MMP), leading to disruption of the host's defense function and degradation of connective tissue and bone matrix [5,6]. In addition, *P. intermedia* produces a protein called interpain-A (INPA), which can modulate the innate immune response and activation of the complement system, initiating the inflammatory process [7]. To reduce this inflammatory process, there is a need for an antimicrobial agent that can inhibit the growth of *P. intermedia* thereby limiting the inflammation and progression of periodontal disease.

Traditional periodontal therapy typically involves mechanical debridement to remove plaque and calculus and the use of antimicrobial agents to reduce the bacterial load [8, 9]. On the other hand, extensive use of antibiotics has resulted in resistant strains, making it imperative to explore alternative antimicrobial strategies. One promising avenue is the use of natural products with inherent antimicrobial properties. Licorice (*Glycyrrhiza glabra*), a perennial herb indigenous to Europe and Asia, has been used in traditional medicine for a long time to treat a variety of illnesses [10]. The root of the licorice plant contains a rich array of bioactive compounds, including glycyrrhizin, glabridin, liquiritin, and flavonoids, which have demonstrated anti-inflammatory, antioxidant, and antimicrobial activities. These properties make licorice an attractive candidate for managing oral infections and promoting periodontal health [11]. This research tends to emphasize the emerging research evidence in favor of the use of natural products in periodontal therapy and to identify potential alternatives to conventional antibiotics that can mitigate the issue of antibiotic resistance.

Depending on the extraction technique and solvent employed, the effectiveness of plant extracts might differ greatly. Two of the most used solvents to eliminate bioactive substances from plants are ethanol and water [12]. Being a polar solvent, ethanol works well for extracting a wide range of phytochemicals, including both lipophilic and hydrophilic substances. This often results in ethanolic extracts possessing a higher concentration of active constituents and, consequently, greater biological activity [13]. Aqueous extracts are more selective for hydrophilic compounds and may not include all of the bioactive ingredients found in ethanolic extracts. Nevertheless, it is widely used in conventional medicine owing to its ease of production and application [14]. Hence, there is a need to establish the antimicrobial potency of both aqueous and alcoholic extracts of licorice against the pathogen. Thus, the present study aims to provide a comparative assessment of the antibacterial effectiveness of ethanolic and aqueous extracts of licorice against the periodontal pathogen *P. intermedia*.

## Materials and methods

Extract preparation

**Figure 1:**
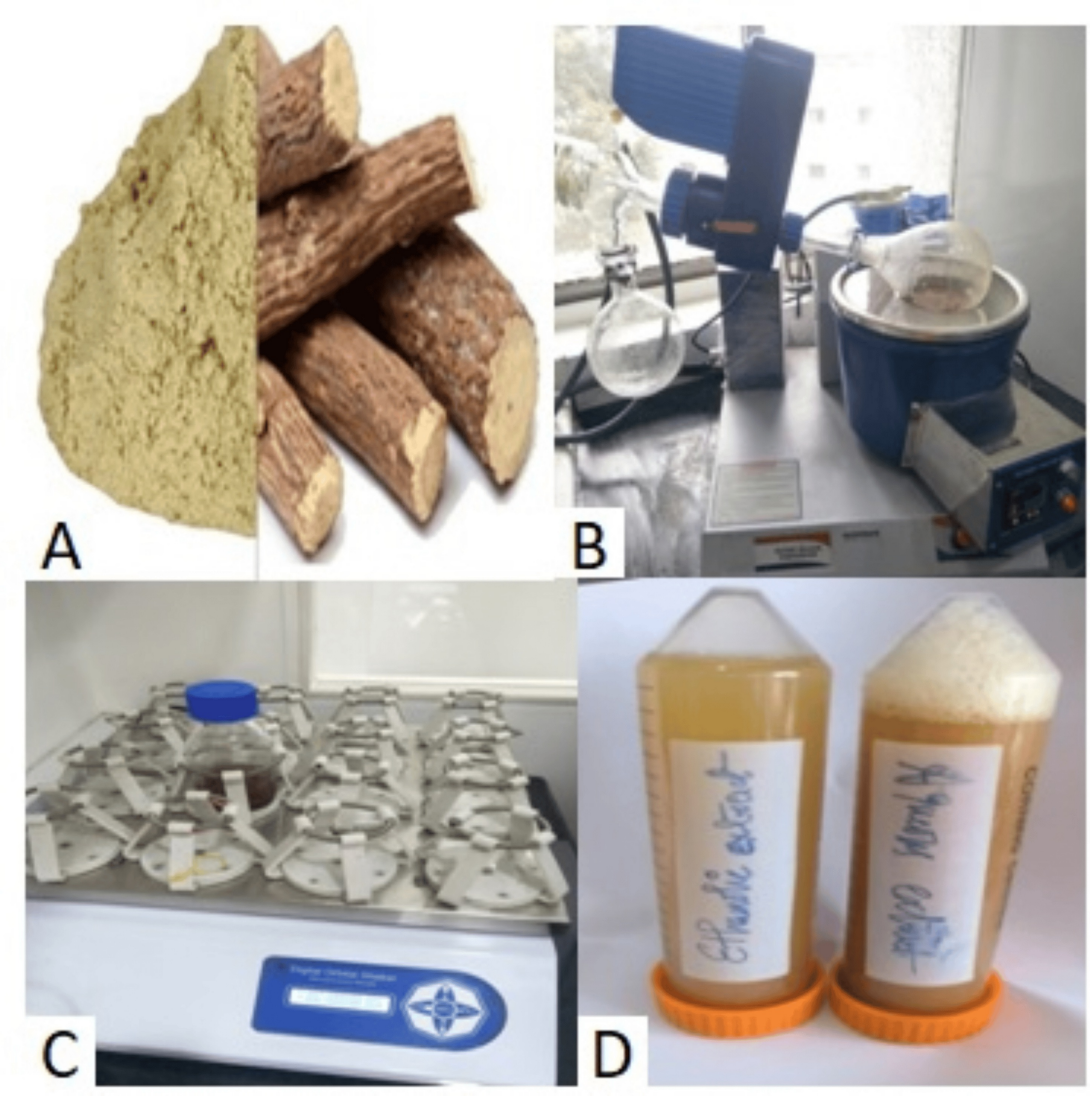
Preparation of aqueous and ethanolic extracts of licorice A: commercially available licorice root powder; B: extract concentrated using a rotary vacuum evaporator; C: extract placed in an orbital shaker; D: prepared extracts stored in an airtight container

Commercially available licorice powder was procured (Indus Valley, Shimla, Himachal Pradesh, India). The aqueous extract was prepared by combining 50 grams of powdered licorice root with 100 milliliters of sterile distilled water in a round bottom flask. The ethanolic extract was prepared when 50 grams of the licorice root powder was macerated in a round bottom flask with 100 milliliters of 70% ethyl alcohol for one week. Both extracts were concentrated by a rotary vacuum evaporator and subjected to occasional shaking to facilitate the extraction process by placing them in an orbital shaker. The resultant mixture was filtered through Whatman No. 1 filter paper to achieve finer filtration. The condensed products were weighed and kept at 4°C before testing. The filtered extract was then transferred to an airtight, amber-colored container for storage, preserving it from light and contamination (Figure 1) [14].

Microbial analysis

The minimum inhibitory concentration (MIC) was evaluated following a standard procedure. Both licorice root extracts (aqueous and ethanolic) were tested against periodontal pathogens. The microbial strain of *P. intermedia* ATCC25611 used in this study, was sourced from Arihant Super Specialty Hospital, Bangalore, Karnataka, India. The bacteria were cultured at 37°C in brain heart infusion (BHI) broth supplemented with 10 µg/mL hemin and 10 µg/mL vitamin K and incubated in an anaerobic environment. For the determination of the MIC in planktonic cells, overnight cultures were diluted in fresh BHI to reach an optical density of 0.2 at 660 nm, corresponding to approximately 10⁶ colony-forming units (CFU)/ml.

**Figure 2:**
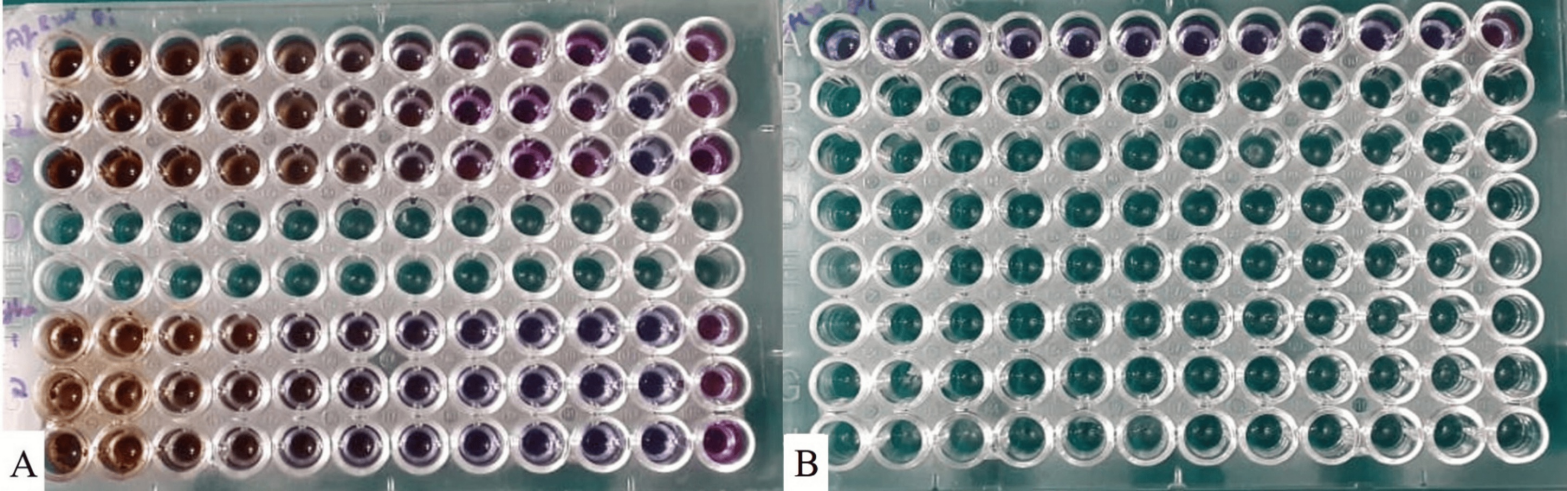
An assay well plate loaded with microorganism and extracts A: an assay well plate loaded with microorganisms along with aqueous extract (top) and ethanolic extract of licorice (bottom), respectively; B: An assay well plate loaded with microorganisms along with chlorhexidine

Sterilized 96-microtitre-plate wells were filled with an equal volume (100µL) of bacterial solution and successive 2-fold serial dilutions of the aqueous and ethanolic extract of licorice (Figure 2). Wells that were treated with microorganisms and broth served as positive controls, and wells with the extract and broth only served as negative controls. Visual documentation of *P. intermedia* bacterial growth was made following a 48-hour incubation period at 37°C. As the lowest concentration at which no discernible bacterial growth occurred, the MIC was calculated. The test was done in triplicates to determine the MIC [14].

## Results

**Figure 3:**
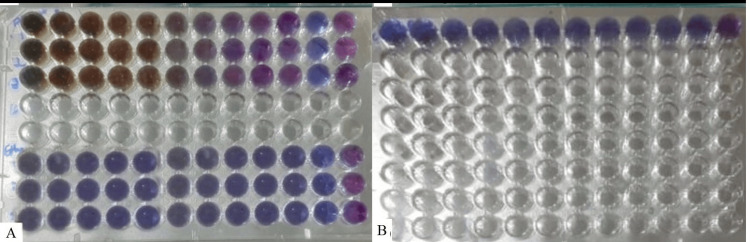
Minimum inhibitory concentration of aqueous and ethanolic extract of licorice against Prevotella intermedia compared to chlorhexidine

In the current study, aqueous and ethanolic extracts of licorice at various concentrations served as test groups. Chlorhexidine served as the control group. In the assay well plate, the red color indicates resistance, and the test color blue indicates the susceptibility of the extract against the microorganism (Figure 3).

Table 1 shows the antimicrobial effect of aqueous licorice root extract of licorice against *P. intermedia* at concentrations of 50%, 25%, 12.5%, 6.25%, 3.1%, and 1.60% and demonstrated resistance to 0.80%, 0.40%, 0.20%, and 0.10% of the extract, respectively. Hence, the MIC of aqueous licorice root extract for *P. intermedia* was established at 0.8% in the current study.

**Table 1:** Minimum inhibitory concentration of aqueous extract of licorice and chlorhexidine against Prevotella intermedia S: susceptible; R: resistant

Groups	50%	25%	12.50%	6.25%	3.13%	1.60%	0.80%	0.40%	0.20%	0.10%
Aqueous extract of licorice	S	S	S	S	S	S	S	R	R	R
	S	S	S	S	S	S	R	R	R	R
	S	S	S	S	S	S	S	R	R	R
Chlorhexidine	S	S	S	S	S	S	S	S	S	S

Table 2 shows the inhibitory effect of ethanolic extract of licorice against *P. intermedia* at concentrations of 50%, 25%, 12.5%, 6.25%, 3.1%, 0.80%, 0.40%, 0.20%, and 0.10%, respectively. Hence, 0.10% is considered to be the least inhibitory concentration of ethanolic extract for *P. intermedia *in the present study.

**Table 2:** Minimum inhibitory concentration of ethanolic extract of licorice and chlorhexidine against Prevotella intermedia S: susceptible; R: resistant

Groups	50%	25%	12.50%	6.25%	3.13%	1.60%	0.80%	0.40%	0.20%	0.10%
Ethanolic extract of licorice	S	S	S	S	S	S	S	S	S	S
	S	S	S	S	S	S	S	S	S	S
	S	S	S	S	S	S	S	S	S	S
Chlorhexidine	S	S	S	S	S	S	S	S	S	S

Table 3 shows an intergroup comparison between the aqueous extract of licorice and chlorhexidine. When the MIC of aqueous licorice extract and chlorhexidine were compared, it was statistically significant (p-value = 0.02).

**Table 3:** Intergroup comparison between the aqueous extract of licorice and chlorhexidine against Prevotella intermedia MIC: minimum inhibitory concentration

Groups	Mean of MIC	95% CI	p-value
Aqueous extract of licorice (test)	1.06±0.6	-0.08, – 2.1	0.02
Chlorhexidine (control)	0.1	0.1	

When the MIC of ethanolic licorice extract and chlorhexidine were compared, both exhibited similar MIC; hence, statistical analysis was not applicable. Thus, ethanolic licorice extract exerted comparable antimicrobial efficacy on par with chlorhexidine.

Table 4 shows the intergroup comparison between the aqueous and ethanolic extracts of licorice. When the MIC of both extracts was compared, it was statistically significant (p-value = 0.0223).

**Table 4:** Minimum inhibitory concentration of aqueous and ethanolic extracts of licorice against Prevotella intermedia MIC: minimum inhibitory concentration

Groups	Mean of MIC	95% CI	p-value
Aqueous extract of licorice	1.06	-.08 – 2.1	0.0223
Ethanolic extract of licorice	0.1	0.1	

## Discussion

Chronic periodontitis is a widely distributed inflammatory disease that involves pathogens like *P. gingivali*s, *A. actinomycetemcomitans*, *T. forsythia*, *F. nucleatum*, and* P. intermedi*a. These bacteria cause disease progression due to virulence factors associated with the pathogenesis [15]. To control and treat periodontal diseases, antibiotics such as tetracycline and mouthwashes such as chlorhexidine were used, and each of them had a few unfavorable side effects. The use of chlorhexidine, which is known as the gold standard, can lead to changes in teeth color and the sense of taste in patients despite its high antimicrobial properties [16]. Thus, there is a need for other natural therapeutic agents that could distinctly target these causative pathogens and can be used as an alternative to conventional chemical plaque control agents. Herbal research is considered a global alternative to overcome these side effects.

*Glycyrrhiza glabra*, the scientific name for licorice, is a member of the Leguminosae family. According to the literature, licorice has attracted the interest of several researchers in recent years. Licorice stems and root extracts are flavonoids that have demonstrated several intriguing biological properties [17]. Licorice extract is found to possess antibacterial activity against different Gram-positive and Gram-negative organisms [18]. Studies have been done previously to identify the antibacterial activity of licorice extract against various oral pathogens, and it has proven to be an effective herbal alternative [19]. However, its effect on specific periodontal pathogens like *P. intermedia* has been explored less [12, 20-21]. This study's findings could pave the way for integrating licorice extracts into oral healthcare regimens, offering a natural and effective means of controlling periodontal pathogens and improving periodontal health outcomes. In herbal preparations, there is a discrepancy in the results related to the efficacy of using aqueous and ethanolic extracts in variable concentrations against pathogens. This variation may be attributed to a better solubility of the active compounds in organic solvents such as methanol and ethanol [22]. Thus, it is necessary to entrench whether the aqueous or ethanolic extract has a potential antimicrobial effect against the pathogen of interest.

In the present in vitro study, we evaluated the antimicrobial activity of both aqueous and alcoholic extracts of licorice against the periodontal pathogen *P. intermedia* and compared its efficacy with chlorhexidine. The findings of the present study showed that licorice extract exhibited antimicrobial activity against* P. intermedia*. Similarly, in vitro studies by Suwannakul and Chaibenjawong [21] and Andrews and Shetti [20] showed that licorice exhibited antimicrobial effects against periodontopathic bacteria. The aqueous extract of licorice showed sensitivity at a mean concentration of 1.06% and the ethanolic extract at 0.1%, respectively. Since, to our knowledge, the present study is the first of its kind, we could not compare the results directly. However, similar to the present study, wherein the effect of licorice was evaluated against other periodontal pathogens conducted by Sharma et al. [12], in their study, aqueous and ethanolic extracts demonstrated antimicrobial effects against *P. gingivalis* at 6.25% and 6.25%, respectively, *F. nucleatum* at 12.5% and 3.12%, respectively, and *A. actinomycetemcomitans* at 0.2% and 0.4%, respectively. Comparing the above results to the present study, it can be inferred that the ethanolic extract exhibited a better magnitude of MIC values than the aqueous extract. The current study demonstrated that, when the aqueous extract and CHX were compared, it showed a significant difference (P = 0.02), showing that the aqueous extract had an antimicrobial effect against *P. intermedia*; however, chlorhexidine was more effective. These findings were in accordance with the results obtained by Ajagannanavar et al. [14]. The ethanolic extract of licorice was sensitive to *P. intermedia* at 0.1% concentration, which is on par with the MIC obtained for chlorhexidine, similar to the study results by Tabatabaeian et al. [23] and Hamdon et al. [24].

Limitations

Within the limitations of the current study, we have explored the antimicrobial properties of licorice with different solvents. However, the current study did not include its effect on fibroblastic activity and cytotoxicity. Such studies can broaden the prospect of using licorice as an alternative agent to chlorhexidine in chemical plaque control.

## Conclusions

In conclusion, the results of the current study indicate that both the aqueous and ethanolic extracts of licorice possess antibacterial activity. Additionally, the present study revealed that ethanolic extract exhibited antimicrobial efficacy at a very low concentration of 0.1% on par with chlorhexidine. Given these findings, licorice can be considered as a potential alternative for chlorhexidine. However, further studies that can explore its other properties, ensuring minimal adverse effects and patient acceptance, should be conducted, which can pave the way for using it as a possible alternative for chlorhexidine mouth rinse in periodontal therapy.
